# Heterogeneous LPS of *Porphyromonas gingivalis* differentially modulate the innate immune response of human gingiva

**Published:** 2011-01-10

**Authors:** TDK Herath, Y Wang, CJ Seneviratne, RP Darveau, CY Wang, LJ Jin

**Affiliations:** 1 The University of Hong Kong, Hong Kong SAR; 2 University of Washington, Seattle, USA; 3 UCLA School of Dentistry, Los Angeles, USA

## Objective

Porphyromonas gingivalis lipopolysaccharide (PgLPS) is a crucial virulence factor strongly involved in chronic periodontitis. PgLPS is known to contain both tetra- (Pg LPS1435) and penta-acylated (PgLPS 1690) lipid A structures with opposing effects. Present study aimed to examine the effect of two Pg LPS isoforms on human gingival epithelium.

## Methods

Reconstituted human gingival epithelia (RHGE) were challenged with two isoforms of PgLPS together with *E. coli* LPS as the positive control. mRNA and proteins were harvested from tissues and culture supernatants were collected. Expression of pro-inflammatory and anti-inflammatory cytokines was evaluated by Q-PCR and ELISA. Involvement of pattern recognition receptors and signaling pathways were also analyzed by Q-PCR and western blot. Next, RHGE was blocked for CD14, TLR2, and TLR4 and followed by stimulation of PgLPS isoforms and effect was evaluated at cytokine level by Q-PCR and ELISA. Furthermore, we used “tissue proteomics” approach to study the differential proteomic expression profiles of gingival epithelium upon Pg LPS stimulation.

## Results

It was shown that penta-acylated PgLPS1690 significantly upregulated the secretion of pro-inflammatory cytokines IL-1β, IL-6, IL-8 and TNF-α in RHGE compared to tetra-acylated PgLPS1435. It seemed that regulation of pro-inflammatory cytokine by PgLPS1690 is mediated through both TLR2 and 4 and CD14/NF-kB axis for most of the cytokines. Proteomic studies indicated a differential protein profiles of RHGE induced with two isoforms.

## Conclusion

*P. gingivalis* LPS heterogeneity differentially modulates the host innate immune response in human gingival epithelium, which may explain the niche-specific pathogenic mechanism of this periodontal pathogen.

